# Mechanisms of Mitral Valve Repair Failure in a Redo Cohort: Association with Patient Characteristics and Initial Repair Features

**DOI:** 10.3390/diseases14090331

**Published:** 2026-09-10

**Authors:** Elisa Mikus, Diego Sangiorgi, Mariafrancesca Fiorentino, Niki Bernardoni, Roberto Nerla, Simone Calvi, Elena Tenti, Fausto Castriota, Carlo Savini

**Affiliations:** 1 Cardiovascular Department, IRCCS Maria Cecilia Hospital, GVM Care & Research, 48033 Cotignola, Italy; 2 Department of Experimental Diagnostic and Surgical Medicine (DIMEC), University of Bologna, 40138 Bologna, Italy

**Keywords:** mitral valve repair or replacement, reoperation, minimally invasive cardiac surgery, surgical technique

## Abstract

Objective: Mitral valve repair failure encompasses heterogeneous anatomical patterns requiring redo surgery, yet the factors determining why repaired valves fail through different mechanisms remain poorly understood. We investigated the association of patient characteristics and index repair features with the anatomical mechanisms of failure among patients requiring redo surgery after degenerative mitral valve repair. Methods: We retrospectively reviewed all consecutive patients undergoing redo mitral valve surgery after previous degenerative mitral valve repair between January 2010 and June 2026. To minimize misclassification bias, only patients with complete operative documentation of the index repair and detailed intraoperative information regarding the mechanism of repair failure were included. Operative reports were reviewed to identify the reconstructive techniques used during the initial repair and the anatomical mechanism of failure at reoperation. To account for mutually exclusive clinical outcomes (prolapse recurrence and mitral stenosis), a competing-risks framework was employed. Results: A total of 121 patients constituted the study cohort. Recurrent leaflet prolapse was the most frequent mechanism of repair failure (71.6%) among patients with mitral regurgitation; mitral stenosis was present in 27.3% overall. Leaflet preservation versus resection, implantation of expanded polytetrafluoroethylene (ePTFE) neochordae, edge-to-edge repair, and annuloplasty ring size were not significantly associated with the anatomical mechanism of repair failure. In multivariable analysis, older age at the index operation was independently associated with recurrent prolapse, whereas younger age and female sex independently predicted the development of mitral stenosis. Within this redo cohort, no statistically significant association was observed between the recorded index repair features and the predominant anatomical mechanisms of failure after adjustment for patient characteristics. Conclusions: Among patients requiring redo surgery after degenerative mitral valve repair, the anatomical mechanisms of failure were associated with selected patient characteristics, whereas no significant association was observed with the recorded index repair features. These findings should be interpreted within the context of a selected redo population and do not support conclusions regarding the comparative durability of different repair strategies.

## 1. Introduction

Degenerative mitral regurgitation is preferentially treated with valve repair rather than replacement, as preserving the native apparatus yields superior long-term survival, better maintenance of left ventricular function, and a lower incidence of procedure-related complications [1,2,3,4]. Over the last thirty years, ongoing advancements in surgical techniques—including leaflet resection, leaflet preservation using expanded polytetrafluoroethylene (ePTFE) neochordae, edge-to-edge repair, and prosthetic annuloplasty—have substantially improved the durability and reproducibility of mitral valve repair, leading to excellent long-term outcomes in experienced centers [1,5,6].

Despite these advances, a small but clinically relevant proportion of patients eventually require reoperation because of recurrent mitral valve dysfunction [7,8]. Repair failure is not a uniform entity but encompasses several distinct anatomical patterns, including recurrent mitral regurgitation due to prolapse recurrence, progressive mitral stenosis, annuloplasty-related complications, and structural alterations of the mitral leaflets or subvalvular apparatus [9,10]. These different mechanisms of failure frequently require different surgical strategies at reoperation and may reflect distinct underlying pathological processes [11,12].

Previous studies have mainly focused on the incidence, durability, and timing of repair failure [13]. More recently, we demonstrated that the interval between the index mitral valve repair and redo surgery is associated with distinct mechanisms of valve dysfunction, with early failures being predominantly characterized by recurrent prolapse and later failures by progressive mitral stenosis and structural leaflet remodeling [9]. These findings suggest that repair failure does not represent a single progressive process but rather a spectrum of different pathological pathways [14,15]. However, the factors determining why an individual repair evolves toward one specific pattern of failure rather than another remain poorly understood.

Both patient-related characteristics and technical aspects of the index repair have been hypothesized to influence the long-term evolution of the repaired valve. Patient age, sex, valve morphology, and the natural progression of degenerative disease may affect tissue remodeling over time. Likewise, different reconstructive strategies—including leaflet preservation or resection, implantation of ePTFE neochordae, edge-to-edge repair, and annuloplasty configuration—modify mitral valve geometry and biomechanics and may contribute to the mechanism through which the repair ultimately fails [16,17]. However, the relative contribution of patient characteristics and index repair features to the anatomical pattern of repair failure has not been systematically investigated.

A better understanding of the determinants of distinct failure patterns may provide new insights into the mechanisms underlying repair durability and contribute to a more individualized follow-up after mitral valve repair. Identifying patients at increased risk for specific modes of repair failure may also facilitate earlier recognition of recurrent valve dysfunction and improve planning for redo surgery.

The aim of the present study was to investigate the association of patient characteristics and index repair features with the anatomical mechanisms of failure among patients undergoing redo surgery after degenerative mitral valve repair. Importantly, given the absence of the denominator of patients undergoing primary repair, the study was not designed to assess the incidence or comparative durability of different repair strategies. Specifically, we evaluated the relative contribution of patient characteristics and index repair features to the different mechanisms of repair failure and explored factors associated with the development of mitral stenosis.

## 2. Materials and Methods

### 2.1. Study Population

We retrospectively reviewed all consecutive adult patients (≥18 years) who underwent redo mitral valve surgery at our institution between January 2010 and June 2026 following a previous surgical mitral valve repair.

Patients were eligible if the index repair had been performed for degenerative mitral valve disease and reoperation was required because of recurrent mitral valve dysfunction. Patients undergoing redo surgery for active or healed infective endocarditis were excluded in order to specifically investigate non-infective mechanisms of repair failure.

During the study period, all consecutive patients undergoing redo mitral valve surgery after previous mitral valve repair were retrospectively screened. To address the study objectives while minimizing misclassification bias, only patients with complete operative documentation of the index repair and detailed intraoperative information regarding the mechanism of repair failure at redo surgery were included. Operative reports from both the index procedure and the redo operation were systematically reviewed to identify the repair strategy adopted during the initial operation and the anatomical mechanism leading to reintervention. Overall, 121 patients fulfilled these criteria and constituted the study cohort.

The index mitral valve repair had been performed either at our institution or at referring centers. Consequently, the denominator of patients undergoing primary mitral valve repair from which these redo procedures originated could not be accurately established. Furthermore, a proportion of patients had undergone additional cardiac surgical procedures before redo mitral valve surgery. Accordingly, the present study was designed to characterize the mechanisms of failure among patients requiring redo surgery rather than to estimate the risk or incidence of failure associated with individual repair techniques.

The study complied with the Declaration of Helsinki and was approved by the local Ethics Committee (Prot. 9689/2019 I.5/186). Informed consent was collected for all patients included in the analysis.

Clinical, echocardiographic, operative, and postoperative variables were retrieved from institutional databases and operative reports. Particular attention was paid to the index mitral valve repair. The following operative variables were collected whenever available:

- Repair strategy (leaflet preservation versus leaflet resection);
- Implantation of expanded polytetrafluoroethylene (ePTFE) neochordae;
- Leaflet resection (posterior, anterior, or bileaflet);
- Edge-to-edge repair;
- Annuloplasty ring implantation and ring size;
- Other adjunctive repair techniques.

At redo surgery, the anatomical mechanism of repair failure was classified according to intraoperative findings as follows:

- Recurrent leaflet prolapse;
- Mitral stenosis;
- Annuloplasty ring dehiscence;
- Leaflet perforation;
- Leaflet retraction;
- Edge-to-edge dehiscence;
- Artificial chordal failure;
- Other mechanisms.

The surgical strategy adopted at reoperation (repeat repair or valve replacement) and the operative approach (median sternotomy or minimally invasive right minithoracotomy) were also recorded.

### 2.2. Definition of Mitral Stenosis

Mitral stenosis was defined based on the preoperative echocardiographic assessment performed before redo mitral valve surgery, with a mitral valve area (MVA) ≤ 1.5 cm^2^, in accordance with current international recommendations [1]. The echocardiographic assessment was based on the available preoperative examination, and the diagnosis was established echocardiographically rather than on intraoperative hemodynamic measurements. The mean transmitral pressure gradient and heart rate at the time of echocardiographic assessment were not systematically available for all patients and therefore were not included in the analysis. Cardiac rhythm at the time of echocardiography was available and was classified as sinus rhythm (N° = 22 (66.6)) or atrial fibrillation (N° = 11 (33.3)).

The distribution of cardiac rhythms was recorded for patients with mitral stenosis. Intraoperative findings at redo surgery were used to confirm the presence of stenotic dysfunction and to characterize the underlying anatomical mechanism of repair failure. Thus, mitral stenosis was identified on the basis of preoperative echocardiographic findings, while intraoperative assessment provided anatomical confirmation and characterization rather than serving as a separate diagnostic criterion.

### 2.3. Study Endpoints

The primary objective of the study was to investigate the factors associated with the anatomical pattern of mitral valve repair failure requiring redo surgery. In particular, we evaluated the relationships among patient characteristics, index repair features, and the mechanisms of repair failure identified intraoperatively.

Secondary objectives included the identification of factors associated with the development of mitral stenosis, the interval between the index repair and redo surgery, the feasibility of repeat mitral valve repair, and early postoperative outcomes.

### 2.4. Statistical Analysis

Following normality assessment via the Shapiro–Wilk test, continuous variables were presented as medians with interquartile ranges (IQR) and compared with the Mann–Whitney test; categorical variables were expressed as absolute numbers and frequencies and compared with Fisher’s exact test.

Time-to-event outcomes were defined starting from the initial intervention. To account for mutually exclusive clinical outcomes (prolapse recurrence and mitral stenosis), a competing-risks framework was employed. Non-parametric Cumulative Incidence Functions (CIF) and multivariable analyses were performed using Fine–Gray subdistribution hazard regression models to identify independent predictors for both outcomes in the presence of competing risks. The model evaluated the relative prognostic impact of surgical techniques and patient baseline characteristics; the considered set of variables was annuloplasty diameter >30 mm, leaflet resection, Goretex chordae, edge-to-edge, age at 1st surgery, and female gender; to enable direct comparison of adjusted hazard ratios (HRs) between the two outcomes, identical multivariable models were fitted for both endpoints. Covariates were selected a priori based on clinical relevance, ensuring consistent adjustment across outcomes; given the absolute number of events in relation to the number of covariates, a mitigated risk of overfitting was present only in the stenosis model. All analyses were performed using R version 4.5.0 (R Foundation for Statistical Computing, Vienna, Austria), with *p*-values < 0.05 considered statistically significant.

## 3. Results

### 3.1. Study Population and Index Repair Techniques

A total of 121 patients fulfilled the inclusion criteria and constituted the study cohort. The median age at redo surgery was 69 years (IQR 58–75), and 85 patients (70.2%) were male. The median left ventricular ejection fraction was 58% (IQR 55–62.8), and the median EuroSCORE II was 4.4% (IQR 2.4–7.5). Baseline demographic and clinical characteristics are summarized in Table 1.

Patients were categorized into two groups according to the leaflet repair strategy adopted at the index operation. The “Respect” group (*n* = 87) included 54 patients who underwent leaflet preservation with ePTFE neochordal implantation, 14 patients who underwent isolated edge-to-edge repair, and 17 patients who underwent isolated annuloplasty ring implantation without any leaflet resection. The “Resect” group (*n* = 34) included 27 patients who underwent leaflet resection alone and seven patients who underwent leaflet resection combined with edge-to-edge repair. Isolated annuloplasty was included in the Respect group because no leaflet resection was performed in these patients; given the small number of patients undergoing isolated annuloplasty, a separate category would have resulted in a limited sample size and reduced the interpretability of subgroup comparisons.

There were no statistically significant differences in preoperative characteristics between the two groups, including EuroSCORE II (*p* = 0.694; Table 1). Furthermore, the groups showed no significant differences in the proportion of edge-to-edge procedures or the implantation of an annuloplasty ring size ≤ 30 mm (18 [20.7%] vs. 12 [35.3%], *p* = 0.235). Overall, the median annuloplasty ring size was 34 mm (IQR 30–34 mm) (Table 2).

### 3.2. Mechanisms of Repair Failure

Among patients with mitral regurgitation, the most frequent mechanism of repair failure was recurrent leaflet prolapse, observed in 63 patients (71.6%). Less frequent mechanisms included annuloplasty ring dehiscence (15.9%), leaflet perforation (8.0%), leaflet retraction (6.8%), edge-to-edge dehiscence (4.5%), and artificial chordal failure (4.5%). Multiple mechanisms occasionally coexisted in the same patients.

The median interval between the index repair and redo surgery was 4.2 years (IQR 1.4–9.3).

### 3.3. Association Between Index Repair Technique and Mechanism of Failure

The relationship between index repair features and the anatomical mechanisms of repair failure is summarized in Table 3.

**Figure 1:**
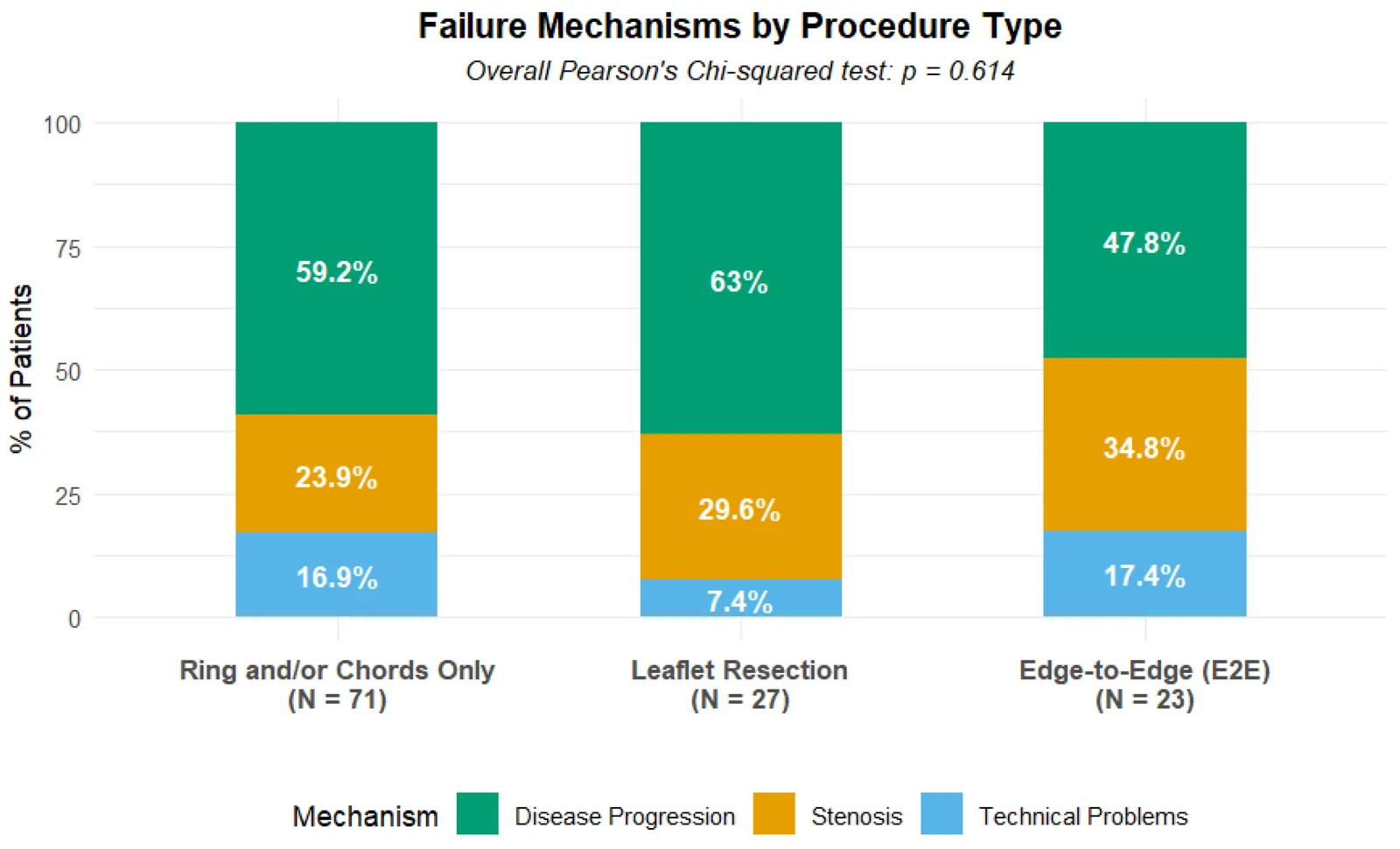
Failure mechanisms by procedure type: technical problems (ring detachment, edge-to-edge detachment); disease progression (prolapse recurrence, leaflet perforation, leaflet retraction, functional regurgitation, chordal rupture).

Overall, the surgical strategy adopted during the index repair was not significantly associated with the mechanism of repair failure (Figure 1). Patients with mitral regurgitation who underwent leaflet preservation and leaflet resection showed comparable rates of recurrent prolapse (66.2% vs. 87.0%, *p* = 0.065), annuloplasty ring dehiscence (20.0% vs. 4.3%, *p* = 0.102), leaflet perforation (7.7% vs. 8.7%, *p* = 1.000), leaflet retraction (9.2% vs. 0.0%, *p* = 0.333), and chordal failure (6.2% vs. 0.0%, *p* = 0.569).

**Figure 2:**
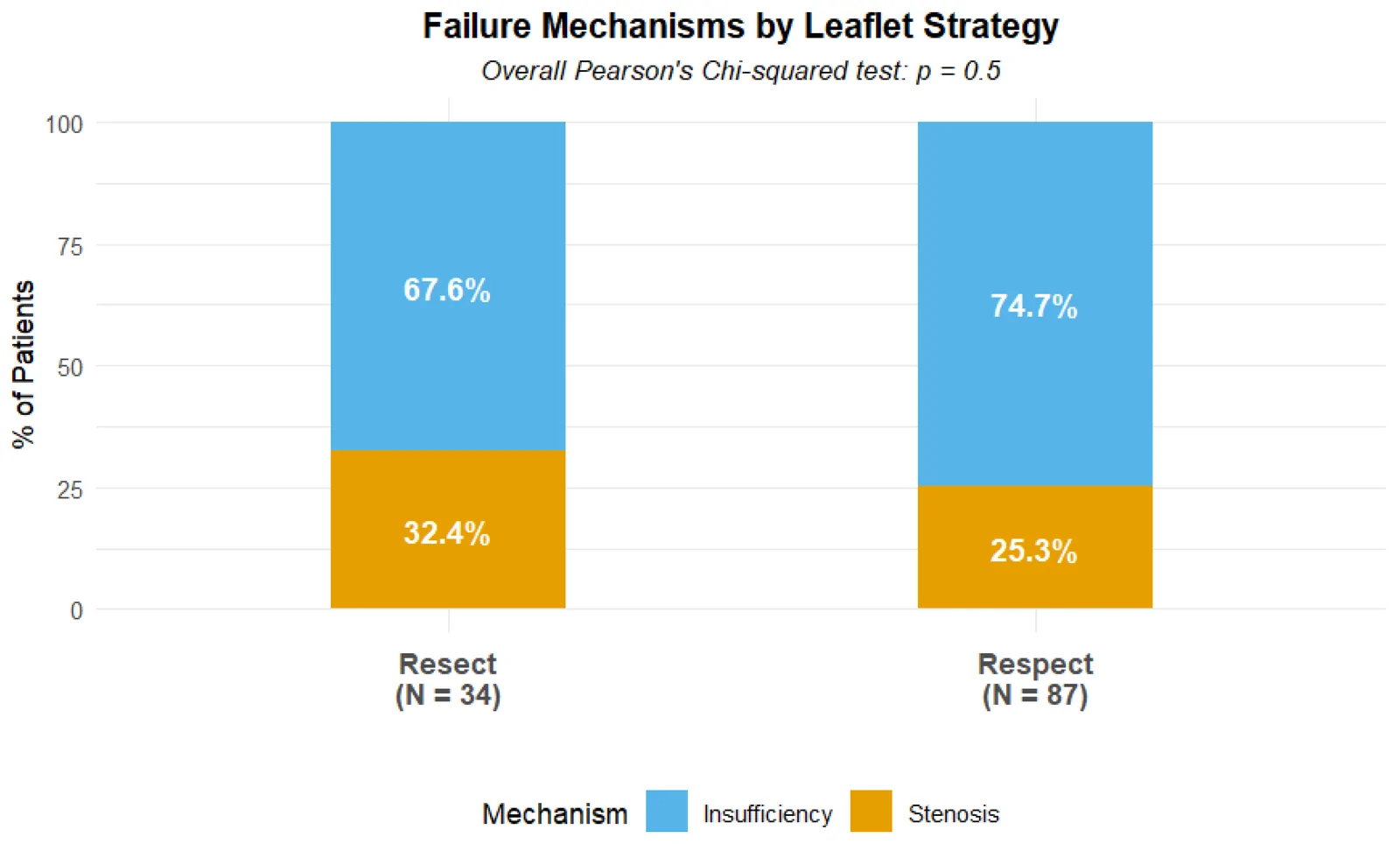
Failure mechanisms according to leaflet strategy.

Similarly, the use of edge-to-edge repair, implantation of ePTFE neochordae, and annuloplasty ring category was not significantly associated with specific mechanisms of repair failure (Figure 2); overall, mitral stenosis was present in 25.3% vs. 32.4%, *p* = 0.577.

### 3.4. Predictors of Mitral Stenosis

**Figure 3:**
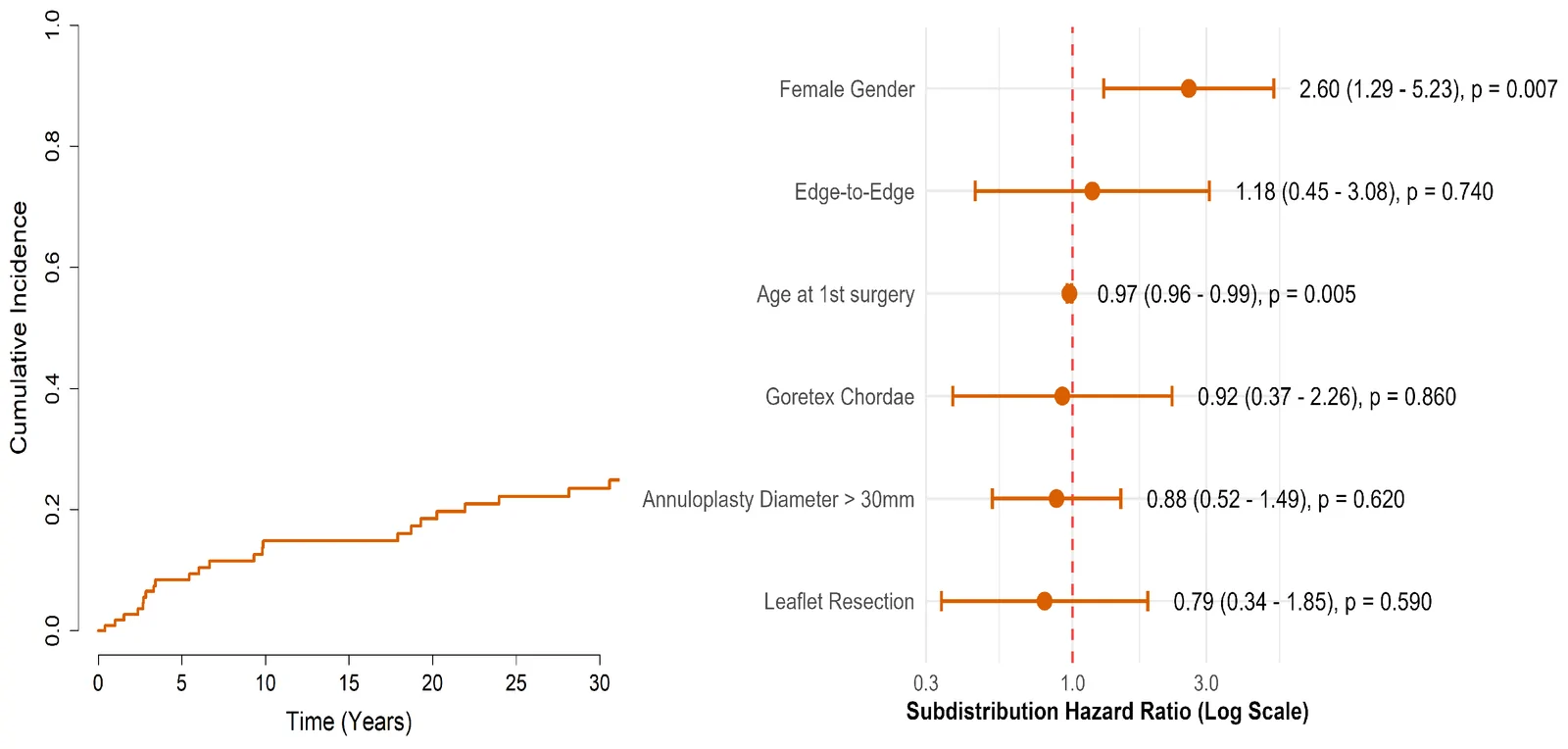
Timing of reoperation for mitral stenosis following index mitral valve repair: cumulative incidence curve (**left**), and forest plot for the Fine–Gray multivariable model (**right**).

The cumulative incidence of reoperation for mitral stenosis, with reoperation for recurrent prolapse treated as a competing event, was estimated using the Fine–Gray method (Figure 3).

The multivariable model identified female sex (HR 2.60, 95% CI 1.29–5.23, *p* = 0.007) as an independent predictor of mitral stenosis, whereas older age at the index operation was protective (HR 0.97, 95% CI 0.96–0.99, *p* = 0.005). No index repair technique remained independently associated with the development of stenotic failure.

Within this selected redo cohort, these findings indicate that older age and female sex are associated with a shorter interval to redo surgery for mitral stenosis, whereas no significant association was observed with the recorded reconstructive strategy.

Overall, these findings indicate that the occurrence of mitral stenosis after repair is predominantly associated with patient-related characteristics rather than with the reconstructive strategy employed during the initial operation.

### 3.5. Predictors of Mitral Prolapse

**Figure 4:**
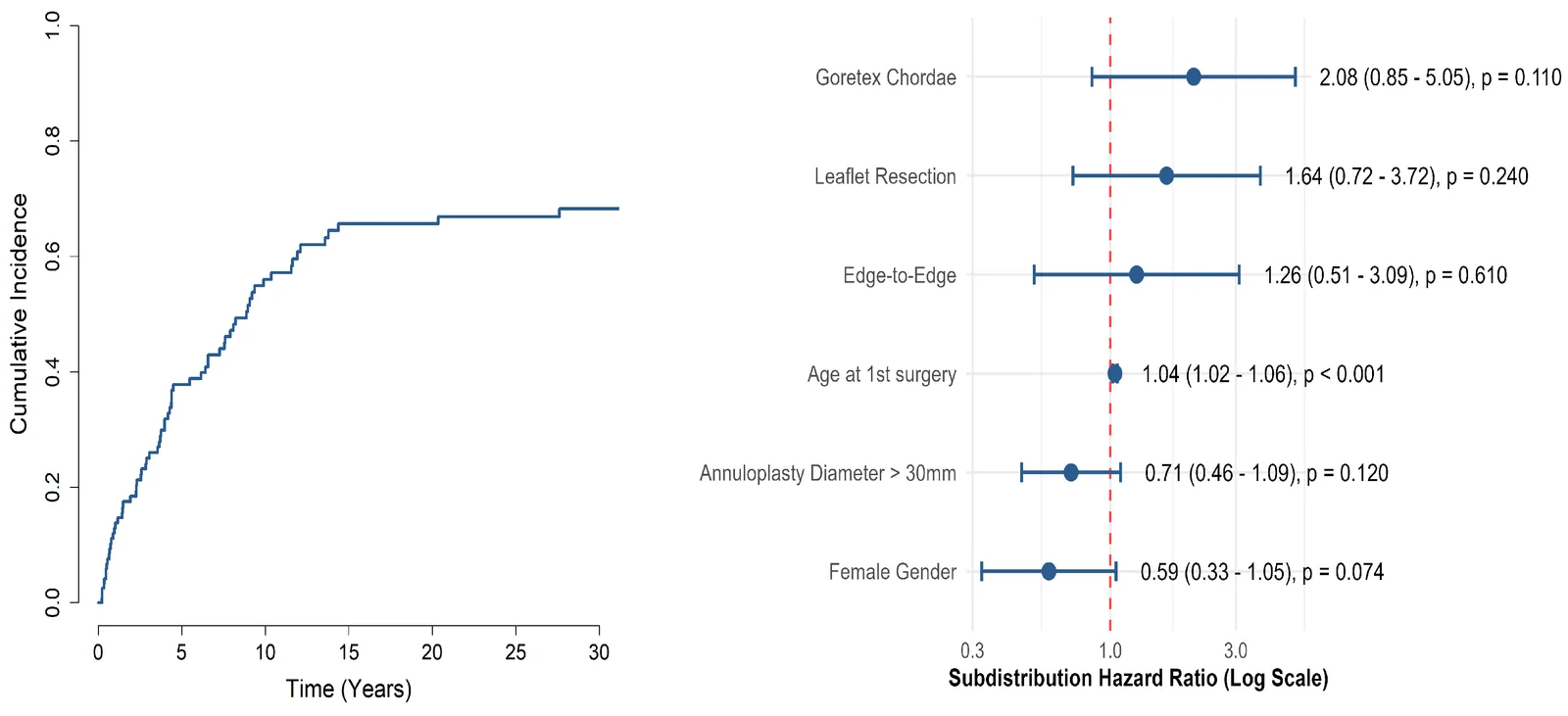
Timing of reoperation for recurrent prolapse following index mitral valve repair: cumulative incidence curve (**left**), and forest plot for the Fine–Gray multivariable model (**right**).

The cumulative incidence of reoperation for mitral prolapse, with reoperation for recurrent stenosis treated as a competing event, was estimated using the Fine–Gray method (Figure 4).

In the multivariable model, older age at the index operation (HR 1.04, 95% CI 1.02–1.06, *p* < 0.001) was independently associated with recurrent prolapse. Conversely, none of the surgical techniques used during the initial repair were independently associated with prolapse recurrence.

These findings suggest that recurrent prolapse after mitral valve repair is more closely related to patient- and era-related factors than to the specific reconstructive strategy adopted during the index procedure.

### 3.6. Surgical Management

Repeat mitral valve repair was performed in 22 patients (18.2%), whereas 99 patients (81.8%) underwent mitral valve replacement.

Overall in-hospital mortality was 1.7%. No significant differences in early postoperative outcomes were observed according to the repair strategy used during the index operation.

## 4. Discussion

### 4.1. Main Findings

The present study investigated the determinants of the anatomical pattern of failure following degenerative mitral valve repair in a contemporary cohort of patients undergoing redo surgery. The principal finding is that, among patients who required redo surgery after previous degenerative mitral valve repair, the recorded reconstructive techniques were not significantly associated with the predominant anatomical mechanisms of failure after accounting for patient characteristics. Selected patient characteristics showed significant associations with the anatomical mechanisms of failure observed within this redo cohort, whereas no significant association was identified between the recorded index repair features and the predominant failure patterns.

Three observations deserve particular attention. First, neither leaflet preservation nor resection, implantation of ePTFE neochordae, edge-to-edge repair, or annuloplasty ring size independently influenced whether repair failure presented as recurrent prolapse or mitral stenosis. Second, older age at the index operation was independently associated with recurrent prolapse and inversely associated with mitral stenosis, whereas female sex specifically predicted stenotic failure. These findings should be considered together with our previous observation that the interval between index repair and redo surgery is associated with different mechanisms of repair failure, with recurrent prolapse predominating among earlier failures and mitral stenosis and structural valve changes becoming more frequent with longer intervals [9]. Third, within this selected redo cohort, selected patient characteristics were associated with the anatomical phenotype and timing of specific failure patterns, whereas no significant association was observed with the recorded reconstructive strategy adopted during the index procedure. However, these findings do not establish that patient-related factors are more important than surgical technique in determining overall repair durability or the risk of failure.

Taken together, these observations provide a different perspective on the mechanisms underlying repair failure requiring reoperation and suggest that patient-related characteristics may contribute to the anatomical phenotype of failure observed at reoperation. These findings should be interpreted within the context of a selected redo population and do not establish the biological evolution of all successfully repaired valves or the relative contribution of patient-related factors and surgical technique to long-term repair durability.

### 4.2. Determinants of Mitral Valve Repair Failure

The durability of mitral valve repair has traditionally been interpreted primarily in relation to the quality of the reconstructive procedure, and previous studies have therefore focused largely on repair techniques and technical predictors of recurrent dysfunction [13,18]. In contrast, the present study addressed a narrower question: among patients who ultimately required redo surgery, which patient characteristics and index-repair features were associated with the anatomical phenotype of failure. Within this selected population, selected patient characteristics were associated with recurrent prolapse and mitral stenosis, whereas none of the recorded index-repair features showed a statistically significant association with these predominant failure mechanisms.

These findings should not be interpreted as evidence that repair strategy does not influence durability. Because the denominator of patients undergoing each repair technique was unavailable, we could not estimate the incidence or relative risk of failure associated with individual techniques or compare their long-term durability. Moreover, the long study period encompassed a progressive shift from leaflet resection towards leaflet-preserving and neochordal techniques, while index procedures were performed both at our institution and at referring centers. Surgical era, institutional setting, and evolving repair philosophy are therefore closely intertwined and represent potential sources of residual confounding.

The timing of failure also provides important context. In our previous study [9], early failures were predominantly characterized by recurrent prolapse, whereas mitral stenosis and structural leaflet changes became more frequent with longer intervals from the index repair to redo surgery. The present findings complement these observations by examining the characteristics associated with the failure phenotype within the redo population. Accordingly, the time-to-event analyses should be interpreted as describing the interval from index repair to reoperation for specific failure phenotypes, rather than as estimates of the absolute incidence or durability of these mechanisms in the overall population undergoing mitral valve repair.

Overall, the absence of significant associations with the recorded repair features should be interpreted cautiously and does not imply equivalence between repair strategies. Rather, among patients who have already progressed to failure requiring surgical reintervention, the anatomical phenotype observed at redo surgery may also reflect patient-related characteristics and the biological evolution of the repaired valve. Further studies including the complete population undergoing primary mitral valve repair, with detailed characterization of repair strategy, surgical era, and institutional setting, are needed to determine the independent contribution of these factors to the incidence and mechanism of repair failure.

### 4.3. Determinants of Recurrent Prolapse

As previously described in the literature [19,20], recurrent leaflet prolapse represented the most frequent mechanism of repair failure, accounting for more than half of all redo procedures. Although different repair strategies modify leaflet geometry in distinct ways, none of the techniques evaluated in the present study independently influenced the occurrence of prolapse recurrence.

Instead, recurrent prolapse was associated with the patient’s age. The association with age may reflect the progressive evolution of degenerative mitral valve disease and age-related changes affecting the native valve tissue over time.

### 4.4. Determinants of Mitral Stenosis

Mitral stenosis represents the second most frequent mechanism of repair failure. Traditionally, post-repair stenosis has been attributed to technical factors such as excessive annular downsizing, leaflet resection, or reduced leaflet mobility [21,22]. Although these mechanisms may contribute to stenotic failure in individual patients, our analysis did not demonstrate an independent association between any specific reconstructive technique and the subsequent development of stenotic failure.

Female sex was independently associated with an increased risk of mitral stenosis, whereas older age at the index operation was independently associated with a lower risk. The association with female sex may reflect anatomical and biological differences that could predispose to a smaller effective mitral orifice after repair, although the mechanisms underlying this finding cannot be established from the present data. The inverse association between age and stenotic failure is less intuitive and should be interpreted cautiously. One possible explanation is that younger patients may have a higher prevalence of Barlow’s disease, characterized by redundant leaflet tissue and more complex valve morphology, potentially requiring more extensive reconstructive procedures. Alternatively, stenotic dysfunction may require a prolonged interval to become clinically manifest through progressive restrictive remodeling, pannus formation, or other structural changes affecting the repaired valve.

Competing mortality may represent another possible explanation for the lower observed risk among older patients, as they may be more likely to die from other causes before stenotic failure becomes clinically evident. However, this hypothesis was not formally evaluated in the present study. Therefore, the observed association between older age and lower risk of stenosis should not be interpreted as evidence of a biological protective effect of age.

Overall, our findings suggest that stenotic failure after mitral valve repair is likely multifactorial and cannot be attributed solely to the reconstructive technique. Further studies incorporating detailed baseline valve morphology, echocardiographic parameters, surgical strategy, and longitudinal imaging are needed to clarify the mechanisms underlying post-repair mitral stenosis.

### 4.5. Relationship with Previous Literature and Clinical Implications

Most previous studies investigating failed mitral valve repair have focused on repair durability, freedom from reoperation, or the timing of recurrent valve dysfunction. Contemporary series consistently identify progression of the underlying degenerative disease as the leading cause of late repair failure, whereas technical failure accounts for only a minority of cases [18,23]. Conversely, other authors have suggested that specific repair techniques, particularly leaflet resection and aggressive annuloplasty downsizing, may predispose to late functional mitral stenosis [24].

Unlike these studies [13,18,24,25,26,27], which primarily addressed when repair fails and whether re-repair should be performed, our study specifically investigated what determines the anatomical mechanism of repair failure. After adjustment for potential confounders, none of the reconstructive techniques evaluated were independently associated with either recurrent prolapse or mitral stenosis. Instead, patient-related characteristics emerged as the principal determinants of the two predominant failure patterns.

These findings complement the existing literature by suggesting that, once a durable repair has been achieved, the long-term evolution of the repaired valve may depend more on the patient’s biological substrate than on the reconstructive strategy itself [28,29,30]. From a clinical perspective, this supports the current practice of selecting the repair technique according to valve anatomy and surgical expertise, while emphasizing the importance of individualized postoperative surveillance. Older patients were at increased risk of recurrent prolapse, mitral stenosis was inversely associated with age, and female sex independently predicted stenotic failure. These associations may represent a hypothesis-generating basis for further studies evaluating whether patient characteristics could help identify individuals who might benefit from more tailored echocardiographic surveillance after mitral valve repair. However, our study was not designed to evaluate surveillance strategies, and these findings should not be interpreted as a recommendation for intensified follow-up.

### 4.6. Limitations

This study has several limitations. First, its retrospective design is inherently subject to selection bias and unmeasured confounding, precluding any causal inference. Although only patients with complete operative documentation of the index repair were included to minimize misclassification bias, residual confounding cannot be excluded.

Second, the study population consisted exclusively of patients undergoing redo mitral valve surgery after previous repair. Consequently, the findings characterize the distribution and correlates of failure mechanisms among patients requiring surgical reintervention and cannot be used to estimate the incidence or comparative durability of individual repair strategies. The absence of a denominator for patients undergoing each index repair technique also prevents meaningful comparison of repair durability between RESPECT and RESECT strategies.

Third, the long study period and the inclusion of index procedures performed both at our institution and at referring centers introduce potential confounders related to surgical era, surgeon experience, institutional setting, and evolving repair philosophy. In particular, the progressive transition from resection towards leaflet-preserving and neochordal techniques limits the ability to disentangle the effects of surgical era and repair strategy. Furthermore, the relatively small number of patients with mitral stenosis and less common failure mechanisms may have limited the statistical power to detect clinically meaningful associations with individual repair techniques. The frequent combination of reconstructive procedures also limits the ability to isolate the effect of individual surgical maneuvers.

Finally, detailed echocardiographic and morphological data at the time of the index repair were unavailable for a substantial proportion of patients, particularly because many index procedures were performed at referring centers or many years before redo surgery. Consequently, variables such as regurgitation severity, specific leaflet segments, Barlow’s disease versus fibroelastic deficiency and annular calcification could not be consistently assessed and were not included in the analysis because of extensive missing data. At redo surgery, mitral stenosis was defined on the basis of the available preoperative echocardiographic assessment, with intraoperative inspection used only to confirm and anatomically characterize the stenotic mechanism. However, the retrospective nature of the study precluded consistent availability of the mean transmitral gradient, so missing parameters were not imputed. Therefore, although mitral valve area was used as the principal criterion for defining stenosis, the absence of complementary haemodynamic information represents a limitation and may have resulted in some degree of misclassification of the stenotic failure phenotype. This limitation should be considered when interpreting the findings regarding mitral stenosis. More broadly, the incomplete echocardiographic and morphological characterization at the index operation prevents a comprehensive assessment of the relationship between the native valve phenotype and the subsequent mechanism of repair failure.

Nevertheless, this study represents a large series of redo mitral valve surgeries with detailed operative documentation of both the index repair and the reoperation.

## 5. Conclusions

Among patients requiring redo surgery after degenerative mitral valve repair, recurrent prolapse and mitral stenosis were the predominant mechanisms of failure. Within this selected redo cohort, these failure phenotypes were associated with patient characteristics rather than with the recorded features of the index repair. These findings do not establish equivalence between repair strategies or their comparative durability, but suggest that patient-related factors and the biological evolution of the repaired valve may contribute to the mechanism of failure once reoperation becomes necessary. Further studies including the full population undergoing primary mitral valve repair are warranted to clarify the independent contribution of patient characteristics, surgical strategy, and surgical era to repair failure.

## Figures and Tables

**Table 1:** Baseline demographic and clinical characteristics.

	Overall	Respect	Resect	*p*
*n*	121	87	34	
Age, median (IQR)	69 (58, 75)	69 (58, 75)	69 (57, 74)	0.940
Male (%)	85 (70.2)	61 (70.1)	24 (70.6)	1.000
BMI, median (IQR)	25.6 (23.1, 28.0)	25.5 (22.8, 28.2)	25.7 (23.6, 27.6)	0.851
Hypertension (%)	73 (60.3)	57 (65.5)	16 (47.1)	0.067
Diabetes (%)	9 (7.4)	8 (9.2)	1 (2.9)	0.442
Dyslipidemia (%)	54 (44.6)	40 (46.0)	14 (41.2)	0.687
Smoking (%)	29 (24.0)	18 (20.7)	11 (32.4)	0.236
Atrial fibrillation (%)	34 (28.1)	24 (27.6)	10 (29.4)	0.826
Pacemaker (%)	7 (5.8)	5 (5.7)	2 (5.9)	1.000
NYHA 3–4 (%)	44 (36.7)	34 (39.5)	10 (29.4)	0.118
LVEF, median (IQR)	58 (55, 63)	57 (54, 63)	60 (55, 61)	0.335
Stroke (%)	5 (4.1)	4 (4.6)	1 (2.9)	1.000
Creatinine, median (IQR)	1.00 (0.82, 1.12)	1.00 (0.81, 1.13)	1.00 (0.83, 1.11)	0.883
COPD (%)	15 (12.4)	11 (12.6)	4 (11.8)	1.000
Isolated mitral valve repair (%)	82 (67.8)	56 (64.4)	26 (76.5)	0.279
EuroSCORE II, median (IQR)	4.41 (2.40, 7.46)	4.46 (2.30, 7.08)	3.53 (2.54, 7.75)	0.694
Minimally invasive (%)	27 (22.3)	22 (25.3)	5 (14.7)	0.236
CPB time min, median (IQR)	118 (90, 141)	118 (95, 143)	118 (81, 132)	0.426
Cross-clamp time min, median (IQR)	86 (68, 110)	86 (72, 111)	84 (64, 109)	0.543
Re-repair (%)	22 (18.2)	19 (21.8)	3 (8.8)	0.119

BMI: Body Mass Index; COPD: Chronic Obstructive Pulmonary Disease; CPB: Cardiopulmonary Bypass; IQR: Interquartile Range; LVEF: Left Ventricular Ejection Fraction; NYHA: New York Heart Association.

**Table 2:** Index repair features.

	Overall	Respect	Resect	*p*
*n*	121	87	34	
Annuloplasty diameter (%)				0.177
no	10 (8.3)	8 (9.2)	2 (5.9)	
26 mm	1 (0.8)	0 (0.0)	1 (2.9)	
28 mm	12 (9.9)	8 (9.2)	4 (11.8)	
30 mm	17 (14.0)	10 (11.5)	7 (20.6)	
32 mm	16 (13.2)	8 (9.2)	8 (23.5)	
34 mm	36 (29.8)	28 (32.2)	8 (23.5)	
36 mm	18 (14.9)	16 (18.4)	2 (5.9)	
38 mm	9 (7.4)	7 (8.0)	2 (5.9)	
40 mm	1 (0.8)	1 (1.1)	0 (0.0)	
42 mm	1 (0.8)	1 (1.1)	0 (0.0)	
ePTFE neochordae (%)	54 (44.6)	54 (62.1)	0 (0.0)	<0.001
Edge-to-edge (%)	23 (19.0)	16 (18.4)	7 (20.6)	0.800

ePTFE: expanded Polytetrafluoroethylene. Categories are not mutually exclusive; columns reflect overlapping procedural components (2 patients in the Respect group underwent combined edge-to-edge repair, leading to a total of 16 cases).

**Table 3:** Relationship between index repair features and the anatomical mechanisms of repair failure.

	Overall	Respect	Resect	*p*
*n*	121	87	34	
Years from 1st surgery, median (IQR)	4.17 (1.42, 9.31)	3.65 (1, 9.14)	5.81 (2.27, 10.10)	0.110
Mitral stenosis (%)	33 (27.3)	22 (25.3)	11 (32.4)	0.577
Mitral regurgitation (%)	88 (72.7)	65 (74.7)	23 (67.6)	0.577
Ring detachment (%)	14/88 (15.9)	13/65 (20.0)	1/23 (4.3)	0.102
Prolapse recurrence (%)	63/88 (71.6)	43/65 (66.2)	20/23 (87.0)	0.065
Leaflet perforation (%)	7/88 (8.0)	5/65 (7.7)	2/23 (8.7)	1.000
Edge-to-edge detach (%)	4/88 (4.5)	3/65 (4.6)	1/23 (4.3)	1.000
Leaflet retraction (%)	6/88 (6.8)	6/65 (9.2)	0/23 (0.0)	0.333
Functional regurgitation (%)	1/88 (1.1)	1/65 (1.5)	0/23 (0.0)	1.000
Chordal rupture (%)	4/88 (4.5)	4/65 (6.2)	0/23 (0.0)	0.569
Failure ePTFE neochord (%)	8/88 (9.1)	8/65 (12.3)	0/23 (0.0)	0.105

ePTFE: expanded polytetrafluoroethylene; IQR: interquartile range.

## Data Availability

The data presented in this study are available on request from the corresponding author. The data are not publicly available due to the Data Protection Directive 95/46/EC.

## Abbreviations

The following abbreviations are used in this manuscript:

BMI	Body Mass Index
COPD	Chronic Obstructive Pulmonary Disease
CPB	Cardiopulmonary Bypass
ePTFE	expanded Polytetrafluoroethylene
IQR	Interquartile Range
LVEF	Left Ventricular Ejection Fraction
NYHA	New York Heart Association.
